# From Defense to Disease: NADPH Oxidase in Cellular Function and Dysregulation

**DOI:** 10.1155/jimr/2847417

**Published:** 2026-06-26

**Authors:** Ankush Prasad, Renu Kushwaha, Phaniendra Alugoju, Pavel Pospíšil, Deepak Rathi

**Affiliations:** ^1^ Department of Biophysics, Faculty of Science, Palacký University, Šlechtitelů 27, Olomouc, 779 00, Czech Republic, upol.cz

**Keywords:** cell differentiation, human diseases, inflammation, innate immune response, macrophage, mononuclear phagocytic system, NADPH oxidases, neutrophils, NOX2, NOX4, oxidative stress, protein oxidation, reactive oxygen species

## Abstract

Nicotinamide adenine dinucleotide phosphate hydrogen (NADPH) oxidase is an important family of enzymes that produce reactive oxygen species (ROS) and consists of NOX1‐5, DUOX1, and DUOX2. These enzymes exhibit diverse tissue distributions with different activation mechanisms involved. They play vital roles in several physiological processes, such as defense against pathogens, wound healing, regulation of gene expression, post‐translational modification of proteins, cell signaling, and cell differentiation. The present review highlights an indispensable role of NOX expression and resulting ROS production on various biological processes, specifically focusing on cell differentiation at various stages of embryonic development and differentiation of monocytes to macrophages within the mononuclear phagocytic system. It also discusses the influence of altered expression of NOX isoforms on the fate of macrophage polarization either towards pro‐inflammatory (M1) or anti‐inflammatory (M2) macrophages. Additionally, it discusses how the dysregulation of the cellular expression of NOXs is involved in the development and progression of a variety of chronic human diseases including cardiovascular diseases, cancer, diabetes, neurological diseases, and autoimmune disorders.

## 1. Nicotinamide Adenine Dinucleotide Phosphate Hydrogen (NADPH) Oxidases

NADPH plays a crucial role in the synthesis of complex molecules by acting as a reducing agent. The active enzyme NADPH oxidase (NOX) transfers electrons from NADPH in the cytoplasm to molecular oxygen (O_2_) in the extracellular space or phagosomal lumen, resulting in the formation of superoxide anion radical (O_2_^●−^) which is then converted into hydrogen peroxide (H_2_O_2_) by dismutation [1]. NOX is an enzyme complex present in the membranes of different cells and organelles, such as mitochondria, the endoplasmic reticulum, the nuclear membrane, etc. [2]. The flavoprotein enzyme family NOX is widely studied in different tissues and cells, more exclusively in phagocytes [3].

The NOX family of enzymes consists of seven members, or isoforms: NOX1‐5, dual oxidase 1 (DUOX1), and dual oxidase 2 (DUOX2). NOX1‐5 consists of six transmembrane domains, and DUOX1 and 2 consists of seven transmembrane domains. The localization and expression levels of these different isoforms vary across tissues. The activation of these different isoforms can have a different mechanism, such as NOX1‐4 requiring a transmembrane subunit protein, p22phox. The complex of NOX1‐4 with p22phox is referred to as cytochrome b558 [4]. In monocytes and macrophages (differentiated forms of monocytes), NOX4 and NOX2 are reported to be present [5].

### 1.1. Assembly and Activation of NOXs

**Figure 1:**
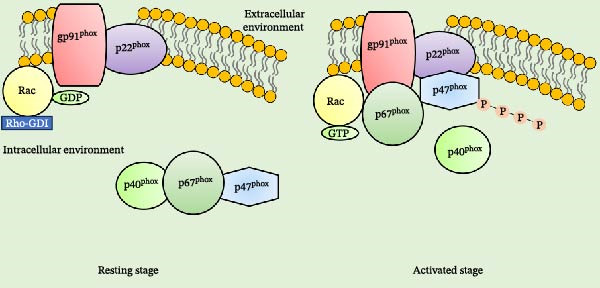
The activation of NAPDH oxidase (isoform NOX2). In the resting stage, membrane bound subunits gp91phox (glycosylated 91‐kDa β‐subunit encoded by CYBB gene) and p22phox subunit (a non‐glycosylated 22‐kDa α‐subunit encoded by CYBA gene) form a heterodimeric flavocytochrome b558, an inactive complex. The cytosol consists of three Phox proteins, p47, p67, and p40 that form a heterotrimer and the GDP bound G‐protein Rac which is stabilized by RhoGDI (Rho GDP‐Dissociation Inhibitor). Upon activation, cytosolic subunits including Rac translocate to the membrane and form an active complex with the flavocytochrome b558. The active complex enzyme generates O_2_^●−^ by accepting electrons (*e* ^−^) from cytoplasmic NADPH and donating it to the molecular oxygen (O_2_).

NOX assembly and activation are tightly regulated processes that involve the translocation of cytosolic components (p47phox, p67phox, p40phox, and Rac GTPase) to the membrane‐bound core (gp91phox/NOX2 and p22phox). Under unstimulated conditions, the NOX subunits remain unassembled: the core complex (flavocytochrome b558, composed of gp91phox and p22phox) is located in the membrane, while the cytosolic components reside in the cytosol. The initiation of the NOX assembly, which precedes its activation in phagocytes, starts with the detection of a chemoattractant. These activation signals initiate intracellular signaling via different pathways such as protein kinase C (PKC), mitogen‐activate protein kinase (MAPK), and phosphoinositide 3‐kinase (PI3K) pathways. After the detection of the chemoattractant or activation signal, the phosphorylation of p47phox Ser345 (pSer345) occurs [3]. This triggers a conformational change exposing its phox homology domain (PX domain) and SH3 domain. This step is followed by the translocation of the phosphorylated cytosolic subunit to the membrane. p47phox anchors the complex to the membrane by binding Phosphatidylinositol 4,5‐bisphosphate (PIP2) via its PX domain and to p22phox via its SH3 domain [3, 4]. P67phox and p40phox are recruited to the membrane by the interaction with p47phox and other proteins. The p22phox and p47phox complex provides a platform for the binding of the p67phox and p40phox complex, whereby p67phox also has a binding site for NOX, a subunit of the membrane complex. For the formation of the fully active complex, Rac GTP binding to the assembled complex is required. Thus, activation of Rac GTPase by guanine nucleotide exchange factors (GEFs) converts Rac from its inactive GDP‐bound state to an active GTP‐bound state, which then binds to the pre‐assembled cytosolic complex and interacts with gp91phox. The interaction of the cytosolic complex with the membrane complex converts the inactive complex into the active complex. This fully active complex, comprising flavocytochrome b558, p47phox, p67phox, and p40phox, together with Rac1, initiates electron transfer from NADPH and facilitates the production of superoxide anion radical (O_2_^●−^) [6, 7] (Figure 1).

While NOX2 requires stimulus‐dependent assembly of cytosolic subunits (p47phox, p67phox, and p40phox) and Rac‐GTPase, both NOX1 and NOX3 depend on NOXO1/NOXA1 homologs. NOX4 is constitutively active and predominantly releases H_2_O_2_. NOX5 and DUOX enzymes are activated by Ca^2+^ binding to EF‐hand motifs [3]. NOX enzymes produce compartmentalized ROS signals through the integration of a single electron‐transfer mechanism and isoform‐specific regulatory modules, aiding host defense, redox signaling, and pathological conditions such as fibrosis and vascular dysfunction. In contrast to other cellular oxidoreductases, NOX enzymes focus on producing ROS instead of metabolizing them, emphasizing their potential as therapeutic targets [8].

### 1.2. NOX Isoforms

The primary function of NOXs is the regulated generation of ROS, which play essential roles in host defense, cell signaling, and differentiation. To date, seven NOX family members have been identified in mammals: NOX1, NOX2, NOX3, NOX4, NOX5, and the dual oxidases DUOX1 and DUOX2 [2, 9]. Although all NOX isoforms catalyze the transfer of electrons from NADPH to O_2_, they differ markedly in tissue distribution, regulatory subunits, and activation mechanisms.

NOX1 and NOX2 are the best‐characterized isoforms in immune cells. NOX2 (also known as gp^91phox^) is highly expressed in phagocytes, including monocytes and macrophages, where it plays a central role in antimicrobial defense through the generation of O_2_^●−^ during the respiratory burst. In addition to host defense, NOX2‐derived ROS have been implicated in redox‐dependent signaling pathways that influence monocyte‐to‐macrophage differentiation, polarization, and inflammatory responses. However, the precise contribution of NOX2 to macrophage lineage remains incompletely understood and appears to be context‐ and stimulus‐dependent [2, 10].

**Figure 2:**
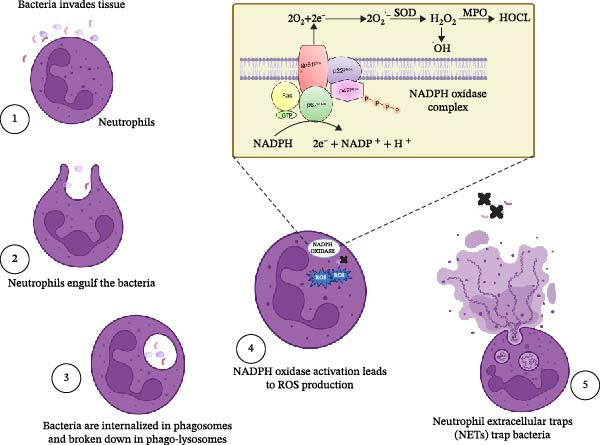
Mechanism of neutrophil‐mediated bacterial killing and NETosis: Steps involved in neutrophils against bacterial infection and the role of NADPH oxidase: Neutrophils recognize and phagocytose bacteria, forming a phagolysosome. Activation of NADPH oxidase is responsible for the production of reactive species such as superoxide anion radical, hydrogen peroxide, and hypochlorous acid. These ROS collectively mediate intracellular bacterial killing by damaging bacterial biomolecules. Additionally, neutrophils may undergo NETosis, a process involving the release of neutrophil extracellular traps (NETs), which consist of DNA‐based structures that trap pathogens.

Correspondingly, NOX2 facilitates ROS production in neutrophils during pathogen invasion. During infection, activated neutrophils generate O_2_^●^− via the NOX2. The O_2_^●−^ is subsequently converted into H_2_O_2_ and other ROS species with the contribution of enzymes such as myeloperoxidase (MPO) [11] (Figure 2). Moreover, ROS generation through the NADPH‐dependent pathway promotes subsequent activities, including the intensified release of neutrophil extracellular traps (NETs) and enhanced production of pro‐inflammatory cytokines [12].

NOX1 is expressed at lower levels in monocytes and macrophages but is upregulated during differentiation and activation. Emerging evidence suggests that NOX1‐derived ROS may contribute to macrophage differentiation and inflammatory signaling, particularly through the modulation of MAPK, NF‐κB, and STAT pathways. Nevertheless, the specific and potentially non‐redundant roles of NOX1 versus NOX2 in macrophage biology remain elusive and require further investigation [13]. Circulating monocytes are highly plastic precursors capable of differentiating into distinct macrophage subsets in response to environmental cues, and NOX‐dependent redox signaling is increasingly recognized as a regulator of this process [10, 14].

Other NOX isoforms exhibit distinct biological features. NOX3 is predominantly expressed in the inner ear and is mainly involved in otoconia formation. NOX4‐derived ROS are primarily linked to differentiation, cellular senescence, and fibrotic signaling rather than acute inflammatory responses. NOX5, DUOX1, and DUOX2 are calcium‐dependent isoforms that do not require cytosolic organizer subunits; DUOX enzymes are particularly important in epithelial tissues, where they contribute to barrier function, host defense, and redox‐mediated signaling [2].

Activation of NOX can be triggered by multiple upstream pathways, including direct activation via PKC. PKC is not a single enzyme but a family of at least 10 serine/threonine kinase isotypes, classified into conventional, novel, and atypical PKCs based on their cofactor requirements [15, 16]. PKC‐mediated phosphorylation of cytosolic NOX subunits (such as p47^phox^) promotes their translocation to the membrane and the assembly of the active enzyme complex, thereby stimulating ROS production. Studies using selective PKC inhibitors from the indolocarbazole and bisindolylmaleimide classes have provided strong evidence for PKC involvement in NOX activation; however, the relative contribution of individual PKC isotypes to NOX regulation in monocytes and macrophages remains incompletely understood [17].

## 2. Cell Differentiation and NOX Expression

### 2.1. Cell Differentiation Types and Their Significance

In multicellular organisms, during early embryonic development, cells appear as totipotent stem cells, which can form different types of cells or even an entire embryo. Indeed, these totipotent cells progressively pass through several developmental stages to form a specialized cell type, which could be both embryonic and extra embryonic cells [18, 19]. As the development progresses, totipotent cells get transformed into pluripotent stem cells, which form the basis for the development of different organs (but not extra embryonic tissues) and functional organisms [20]. Pluripotent cells subsequently differentiate into multipotent, oligopotent, and unipotent cells. The unipotent stem cells still retain their self‐renewal capacity and can further differentiate into specialized cells like monocytes [21]. Many cellular proteins contribute to cell differentiation, cell growth, and cell development [22, 23]. NOX enzymes are known to be vital regulatory proteins responsible for the generation of ROS involved in the cell differentiation process [24].

### 2.2. Cell Differentiation and Its Correlation With NOX Expression

The NOX enzymes are involved in various physiological functions such as cellular defenses, wound healing, and gene expression regulation [25]. Their expression varies in different stem cells. For example, embryonic stem cells show low expression of the NOX enzymes, which is required for the maintenance of low levels of ROS [26]. Stem cells derived from perinatal tissues, such as chorion and amnion, particularly the amniotic stem cells, can differentiate into all three germ layers. It has been reported that placental amnion cells exhibit high expression of NOX and a concomitant rise in ROS, which are presumed to be essential for the angiogenesis in the placenta [27]. The expression of various NOX isoforms at different stages of embryonic development was studied in the zebrafish model (Table 1). Additionally, research findings from several labs revealed that NOX enzymes, such as NOX2 and NOX4, can influence the self‐renewal capability of murine‐induced pluripotent stem cells (iPSCs) [28–31].

**Table 1:** Stem cells, their differentiation potential, and expression of various NOX isoforms.

Stem cells	Classification	Differentiated potential	NOX expression	Model organisms/sample source	Refs.
Totipotent stem cells	Cells having ability to give rise a whole organisms [19]	Embryo and extra‐embryonic structure [19]	—	—	—
Pluripotent stem cells	Cells can give rise to three germ layers but not extraembryonic structure. Example embryonic stem cell and induced pluripotent stem cells (iPSCs) (20)	Ectoderm, mesoderm and endoderm [19]	NOX1, NOX5, and DUOX expression fluctuation in early 2 days, while *NOX*2/cybb expression remains constant.	Zebrafish embryo	[28]
			NOX2 and NOX4 partially regulated the self‐renewal ability and maintained the pluripotency in murine‐induced stem cells.	Mouse (Murine induced pluripotent stem cells (miPSCs)	[29]
			Knockdown and knockout of the p22^phox^ (NOX1–NOX4) decreased the reprograming.	Mouse (mouse embryonic fibroblasts)	[30]
			In embryonic stem cells, the reduction of the p38α kinase led to upregulated expression of NOX2. Studies suggest that there might be a link between stem cell maintenance and cell differentiation.	Mouse (mouse embryonic stem cells)	[31]
Multipotent stem cells	Having lesser differentiation capacity as compared to the pluripotent stem cells [19]	They could differentiate into a particular type of cell type in the specific tissue and organ [19]	—	—	—
Hematopoietic stem cells (HSCs)	Belongs to the multipotent stem cells [19]	Capable for giving rise to the various types of blood cells [19]	NOX4 participated in the HSCs differentiation form the iPSCs.	Human (human induced pluripotent stem cells)	[32]
			HSCs express the catalytic subunit of NOX1, NOX2, and NOX4. As well as some regulatory subunits p22, p40, p47, p67, rac1, rac2, and NOX other isoforms such as NOXO1, NOXA1 and spliced variant NOX2s.	Human (bone marrow derived hematopoietic stem cells)	[33]
Epithelial stem cells	—	—	A study found that the Hematopoietic cells and progenitor cells express the NOX1, NOX2, and NOX4.	Mouse	[34]
			The deficiency of the NOX2 in Hematopoietic cells/progenitor cells decreased their motility.	Mice	[35]
Neural stem cells	Belongs to the multipotent stem cells [19]	Responsible for the renewal of the epithelial tissue such as intestine, epidermis, mammary gland, and cornea [36]	In colonic stem cells (CSCs), reduced cell proliferation was seen in the absence of the NOX1.	Mice	[37]
			NOX1 depletion and TNFα stimulation in cells led to colitis generation due to disturbance in stem cell environment and cell differentiation.	Mice and human	[38]
	These are self‐renewing multipotent stem cells [39]	Responsible for the generation of nervous system components such as the neurons and glial	Studies concluded that the NOX‐2 derived ROS are required for proliferation and neuronal production in the brain.	Mice, mice and human cells, Neural stem cells isolated from mice pup	[40–42]
			ROS generated through the NOX‐2 in adult hippocampal stem/progenitor cells is crucial for maintaining normal proliferation and regulating their intracellular signaling.	Mouse	[40]
Mesenchymal stem cells (MSCs)	These are the stromal cells having self‐renewal capability [43]	Give rise to various stromal cell lineage [44]	Increase in the intracellular ROS level via NOX4 mediates adipocyte differentiation in MSC.	Rats (rat bone marrow‐derived mesenchymal stem cells)	[45]
Unipotent stem cell	Cells, which are capable for give rise a specific types of cell [19]	—	—	—	—

Abbreviations: CSCs, colonic stem cells; ESCs, embryonic stem cells; HSC, hematopoietic stem cells; iPSCs, induced pluripotent stem cells; MSCs, mesenchymal stem cells.

On the other hand, adult stem cells are multipotent and can differentiate into different tissues and organs. The adult stem cells include hematopoietic stem cells (HSCs), mesenchymal stem cells (MSCs), intestinal stem cells, neuronal stem cells, epithelial stem cells, epidermal stem cells, hair follicular stem cells, and sebaceous gland stem cells [36, 39, 46].

The expression of NOXs in HSCs links their role in the modulation of ROS‐mediated signaling, indicating their crucial role in growth and development. The expression of the catalytic subunit of distinct NOX isoforms (NOX1, NOX2, and NOX4) at the transcription and translation levels was confirmed by Piccoli et al. [33]. Prieto‐Bermejo et al. [34] study analyzed the impact of NOX with deletion of p22phox on in vivo hematopoiesis. It has been demonstrated that the deletion of NOX2 impedes migration of hematopoietic stem/progenitor cells [34, 35]. Brault et al. [32] demostrated that NOX4 is the key enzyme involved in the initial phase of differentiation process from the iPSCs. Previous studies have also reported the involvement of NOX‐induced ROS in the activation of signaling pathways in pluripotent stem cells like spermatogonial stem cells. For instance, NOX1‐induced ROS have been shown to play a crucial role in the self‐renewal of spermatogonial stem cells by activation of the p38 mitogen‐activated protein kinases (p38 MAPK) and Jun N‐terminal kinase (JNK) pathways [47].

Several other studies demonstrated the crucial role of NOX enzymes in the differentiation of multipotent stem cells derived from the colon and intestine. NOX1 and DUOX2 are primarily expressed in the colon stem cells. Findings of van der Post et al. [37] showed that NOX1‐induced ROS positively influenced colon stem cell proliferation via activating the epidermal growth factor receptor. Additionally, other researchers demonstrated that NOX1‐induced activation of tumor necrosis factor (TNF) led to the production of ROS, which is essential for the intestinal stem cells to maintain their niche [38]. Donval et al. [48] findings revealed that NOX signaling is involved in the proliferation of retinal stem cells by preventing their quiescent behavior. In addition, the inhibition of NOX activity by apocynin has been shown to partially reverse the aging of the MSCs by directly affecting the expression of transcription factors such as Nanog and Octamer binding transcription factor 4 (Oct‐4), indicating the crucial role of Nanog and Oct‐4 in stem cell self‐renewal [49]. Li et al. [50] reported that both NOX2 and NOX4 can stimulate the production of high levels of ROS, which led to the acceleration of angiotensin II‐induced late endothelial progenitor cell (EPC)‐senescence. Both NOX4 expression and ROS‐mediated signaling have also been reported to be important for the differentiation and proliferation of osteoblastic progenitor cells [51]. Several other studies have also investigated NOX’s role in the neuronal stem cells and MSCs. It was concluded that NOX‐derived ROS participate in neuronal proliferation and adipocyte differentiation as well [43–45]. Broadly, various studies support the impact of NOX‐2 on the neuronal stem cells’ proliferation and the maintenance of their intracellular signaling [40–42]. Altogether, findings from different research studies confirm that NOX‐induced ROS production is crucial for maintaining the niche of stem cells and progenitor cell proliferation. However, the production of ROS beyond a certain limit causes detrimental effects on the stem cell niche. Future studies are therefore required to understand mechanisms responsible for ROS production in the control environment by NOX, which could provide additional insights into stem cell proliferation and maintenance.

DUOX1 has a major impact on cell differentiation, particularly through its involvement in ROS production. The main site of expression for DUOX1, which regulates redox signaling and influences several cellular processes, are epithelial tissues. Research demonstrates that the maturation factor DUOXA1 and its precursor DUOX1 regulate the formation of muscle satellite cells. Increased ROS production from DUOXA1 overexpression can prevent muscle stem cells from developing and trigger apoptosis [52]. Airway epithelial cells exhibit high levels of DUOX1 expression, which aids in cellular differentiation and homeostasis. DUOX1’s function in preserving a proper differentiation pathway has been suggested by its association with epithelial malignancies through dysregulation [53]. DUOX1 contributes to immunological defense systems and the manufacture of thyroid hormones. Additionally, it affects the development of epithelial cells in salivary glands and gastrointestinal organs, implicating it in host defense responses [54].

Numerous NOX isoforms are elevated to support specialized cellular tasks during cell differentiation, which is a critical process. NOX1 and NOX2 play a role in the maturation of immune cells and the differentiation of vascular smooth muscle cells. Specifically, NOX2 is abundantly expressed in phagocytes, where it supports oxidative burst responses and innate immunity [55]. Widely expressed in endothelial and epithelial cells, NOX4 contributes to the maturation of cardiac myocytes and fibroblasts. Gene expression and extracellular matrix remodeling are influenced by ROS produced by NOX4, and both processes are critical for differentiation [56]. Vascular and immunological cells contain NOX5, which is controlled by calcium signaling and plays a role in oxidative stress reactions during differentiation [57].

According to Burtenshaw et al. [56], DUOX1 and DUOX2 are primarily involved in the manufacture of thyroid hormones and the differentiation of epithelial cells, especially in the tissues of the gastrointestinal tract and airways.

### 2.3. Monocytes to Macrophages Cell Differentiation

HSCs differentiate into myeloid and lymphoid cell progenitor cells that give rise to different types of blood cells. Myeloid progenitor cells produce platelets, erythrocytes, and white blood cells such as neutrophils, eosinophils, basophils, and monocytes [58]. On the other hand, lymphoid progenitor cells produce both T and B lymphocytes as well as natural killer cells. Of note, monocytes contribute to the immune system by differentiating into dendritic cells and macrophages, which are conventionally known as the second line of defense [59]. Macrophages are components of the innate immune system. They reside in most tissues and organs and play an essential role in defense mechanisms such as pathogen engulfment, clearance of cellular debris, and regulation of immune homeostasis. Macrophages are immune cells that occur locally within almost all tissues and organs throughout the human body [60]. In different organs, macrophages are known by specialized names, such as alveolar and interstitial macrophages in the lungs; Kupffer cells in the liver; dermal macrophages in the skin; osteoclasts in bone; adipose tissue macrophages in the adipose tissue; and microglia in the central nervous system (CNS). In the spleen and lymph nodes, macrophages exist as specialized subsets, including red pulp macrophages, marginal zone macrophages, and subcapsular sinus macrophages [60].

Differentiation of monocytes into macrophages is commonly seen during any inflammatory condition, such as viral infection and tissue injury [61, 62]. However, the literature also reveals that macrophages are not only derived from hematopoietic progenitor stem cells, but during embryonic development, they have been reported to be derived from the yolk sac or progenitors derived from the fetal liver [63].

**Figure 3:**
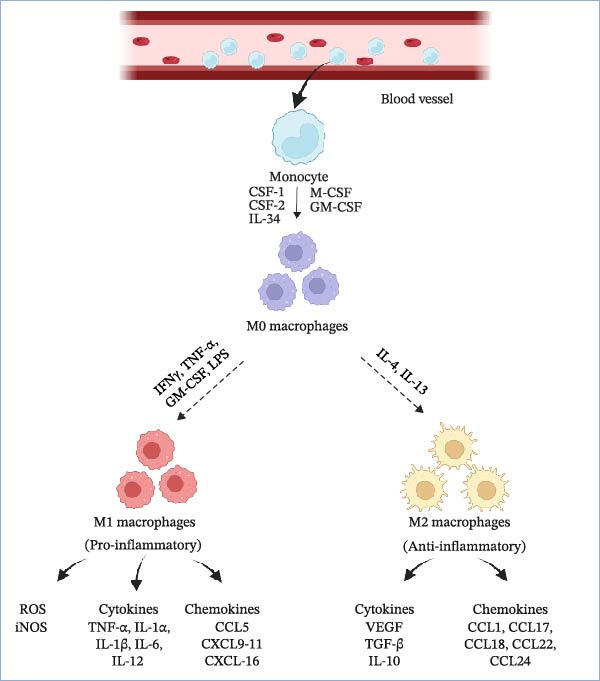
Macrophage differentiation: During pathogen infection or tissue injury, different signaling molecules are released into the blood by pathogens or damaged tissue cells. Upon sensing these signaling molecules, the circulating monocytes migrate to the site of injury or infection, undergo gene expression, change morphology, and differentiate into naive macrophages (M0). During inflammation, an alternative polarization of M0 cells to pro‐inflammatory (M1) or anti‐inflammatory (M2) macrophages occurs. Ex vivo treatment with LPS/IFNγ or IL4/IL13 results in polarization of the cells into M(LPS + IFNγ)/M1 or M(IL4 + IL13)/M2 macrophages, respectively. The M1 macrophages express cell surface specific markers and releases various cytokines, chemokines, and ROS resulting in the activation of the T cell‐mediated defense to destroy the bacteria and viruses. In contrast, M2 macrophages express different cytokines related to various cellular functions. In particular, M2 macrophages play a key role during wound healing and mitigating inflammation.

The monocyte‐to‐macrophage transition is governed by numerous cytokines, growth factors, and signaling pathways [64]. Monocytes differentiate primarily into naive macrophages (M0) in the presence of specific cytokines such as the colony stimulating factor‐1 (CSF‐1), colony stimulating factor‐2 (CSF‐2), and Interleukin‐34 (IL‐34) under in vitro conditions [64] (Figure 3). However, in the natural system, monocyte differentiation is mediated by external stimulus, and cytokines are secreted based on monocyte recruitment. Naive macrophages (M0) are dynamic in nature and can exist in different activation states, termed M1 and M2 macrophages. M0 cells undergo polarization to produce either pro‐inflammatory (M1) or anti‐inflammatory (M2) macrophage phenotypes and alternative activation of these two phenotypes takes place during inflammation. Indeed, the maintenance of constant ratio of M1 to M2 cells is critical for the regulation of homeostasis in vivo. Any alteration in this M1 to M2 ratio can substantially affect severity of inflammation during disease conditions [65]. However, a stable ratio of M1/M2 macrophages is not necessary for homeostasis; rather, the inflammatory process and tissue microenvironment determine how their activation status changes dynamically. Macrophage polarization is generally recognized as a site‐specific phenomenon that occurs locally within almost all tissues and organs, where the local microenvironment and disease progression determine their functional identity.

In the tumor microenvironment, hypoxia or low pH can favor the polarization of tumor‐associated macrophages (TAMs) toward the M2 phenotype, stimulating vascular endothelial growth factor (VEGF) secretion, leading to angiogenesis, and promoting IL‐10 mediated immunosuppression that shields tumors from immune attack. In contrast, modulation of the tumor microenvironment can reprogram TAMs from the M2 phenotype to the M1 phenotype [66].

In spontaneous wound healing, neutrophils are the first immune cells to infiltrate the tissue, followed by recruitment of monocytes that differentiate into macrophages within 48 h. In the early stages after injury, M1 macrophages dominate at the site, where they eliminate pathogens and clear dead cells. During later stages, the environment becomes dominated by M2 macrophages, which promote tissue regeneration and collagen synthesis [67]. In the CNS, during the acute injury phase, microglia (macrophages) polarize toward the M1 phenotype to clear damaged myelin. However, prolonged M1 activation leads to the persistent release of proinflammatory cytokines, which subsequently damages the myelin sheath. The switch toward the M2 phenotype is essential for neuronal repair and myelin restoration [68]. For instance, Guillain–Barré syndrome (GBS) is an autoimmune condition in which macrophage polarization is favored toward the M1 phenotype in the early stage, promoting myelin damage. Later accumulation of M2 macrophages supports anti‐inflammatory signaling and promotes axonal repair [69].

In autoimmune conditions such as rheumatoid arthritis (RA), M1 macrophages predominate in the synovial fluid and joint tissue. The secretion of TNF‐α and IL‐6 by these cells drives bone and cartilage degradation. In contrast, the presence of M2 macrophages facilitates tissue repair [70]. In summary, based on external cues, including cytokines, infections, and tissue injury, macrophages can switch between pro‐inflammatory M1 and anti‐inflammatory M2 states. M1 macrophages predominate during acute inflammation and produce pro‐inflammatory cytokines (IL‐1β, TNF‐α, and IL‐6) that neutralize infections. As the inflammation diminishes, M2 macrophages become more active, facilitating anti‐inflammatory reactions and tissue repair.

The M1 macrophages are triggered by microbial components such as lipopolysaccharide (LPS) and pro‐inflammatory cytokines like interferon‐gamma (IFN‐γ) and TNF‐α. Activation of M1 macrophages leads to the release of chemokines and orchestrates a type I immune response specialized in the elimination of intracellular pathogens and antitumor defense [71]. A defining feature of M1 macrophages is their robust production of ROS and other inflammatory mediators, which facilitates the eradication of intracellular pathogens [72]. Concurrently, anti‐inflammatory macrophages can be subcategorized into M2a, M2b, M2c, and M2d, each expressing distinct signature markers and cytokine profiles. Each M2 subtype plays a specific role in supporting tissue repair, remodeling, and the resolution of inflammation [64]. Although M2 macrophages are pivotal in resolving inflammation and contribute to immunosuppressive responses, they can also play a role in cancer progression [65]. In macrophage dynamics, ROS play a crucial bifunctional role. In M1 macrophages, ROS are derived from NOX (mainly NOX2) and mitochondria and are essential for microbial phagocytic activity. Additionally, ROS serve as central signaling molecules that facilitate the differentiation of M2 macrophages and the development of TAMs [73, 74].

In a study by Xu et al. [14], bone marrow‐derived macrophages were treated with the macrophage‐CSF (M‐CSF) and blocked ROS generation by inhibiting the NOX isoforms NOX1 and NOX2, both individually and in conjunction. This blockage of ROS production results in the inhibition of both extracellular signal‐regulated kinase (ERK) and JNK signaling, subsequently hampering the monocytes to macrophage differentiation [14]. Accumulating evidence shows that macrophage polarization is substantially modulated by the expression of different NOX isoforms. In the following section, we discuss the regulation of macrophage polarization toward either M1 or M2 under the differential expression of NOX isoforms.

### 2.4. Interplay Between NOX Expression, ROS Generation, and Macrophage Polarization

Macrophage polarization is an essential mechanism for controlling the immune response and maintaining cellular homeostasis. ROS are known to be among the key players modulating macrophage polarization. Therefore, several studies have investigated the effects of NOX isoforms on the modulation of macrophage polarization under various experimental settings using multiple in vivo disease model systems [74] (Table 2). Padgett et al. [85] investigated the impact of NOX‐derived O_2_^●−^ on the macrophage morphology in mice affected by delayed type 1 diabetes. Their findings revealed a remarkable influence of NOX‐derived O_2_^●−^ on macrophage differentiation in type 1 diabetes [85]. According to Zhang et al. [73], the treatment of monocytes with butylated hydroxyanisole (BHA) resulted in the inhibition of O_2_^●−^ production and the differentiation of the M2 macrophage cells, without affecting the differentiation of M1. The deletion of Rac1, an essential component of NOX1 and NOX2, strongly confirms the role of ROS‐mediated M2 macrophage polarization. Inhibition of the ROS‐mediated M2 macrophage polarization by BHA also reduced the occurrence of the TAMs, confirming their contribution in the suppression of tumorigenesis [73]. Kumar et al. [79] assessed the impact of the traumatic brain injury (TBI) on the macrophage’s polarization under the influence of ROS. NOX2 expression was found to be increased in proinflammatory macrophages in the TBI model. Furthermore, the deletion/inhibition of NOX2 shifted polarization toward the M2 phenotype via the interleukin 4 receptor (IL‐4R) signaling. Their study findings concluded that the functional inhibition of the NOX2 enzyme modulates the M1/M2 polarization by skewing towards the M2 macrophages, thereby significantly restricting the tissue and neuronal damage [79]. Xu et al. [14] observed that deletion of the NOX1 and NOX 2 resulted in an intense reduction in ROS production, which hampered M2 polymerization but did not have any negative impact on M1 polarization. Furthermore, the deletion of the NOX1–NOX2 significantly reduced the tumor growth and size in the Lewis lung carcinoma cell (LLC) and reduced the prevalence of the M2‐like TAMs [14]. Recently, intercellular communication, or “cross‐talk,” between ROS derived from NOX and mitochondria termed “ROS‐induced ROS release” has been proposed as a mechanism for ROS amplification within distinct subcellular compartments. This process is essential for the activation of redox signaling and may represent a feed‐forward mechanism that promotes localized ROS production [86].

**Table 2:** Macrophage polarization (M1 and M2 macrophages) under the influence of ROS generated by the NOX isoforms.

Model	Inducer/inhibitor	Validation methods	Condition	Protein of interest/markers	NOX isoforms	Macrophages polarization	Signaling	Refs.
WT, NOX1‐KO, NOX2‐KO mice	M‐CSF	PCR, WB, FCM, IF	—	IL‐12β, IL‐1β, TNF‐α, CCL‐17, CCL‐24, INOS, RELM α, F4/80, Caspase1 and Caspase1p20, VEGF, Arginase‐I	NOX1 and NOX2 deletion	Suppressing in M2 macrophages	ERK and JNK signaling	[14]
WT, NOX4^−/−^ and NOXy/‐ mice	LPS/IFN*γ* or IL4/IL13	PCR, WB, FCM, EMSA	Fibrosarcoma	IL1β, TNFα, IL10, ICAM‐1, Ly6C, NFκB, SOD1 or 3, p65, iNOS, arginase 1, MRC1, transglutaminase 2, FIZZ1, YM1, pSTAT6, STAT6, pSTAT1, STAT1, CD45, MMP9, Collagen I, and III	NOX 4 deletion	Towards M1 macrophages	NF*κ*B and STAT6 signaling	[75]
					NOX2 deletion	Reduced M1 macrophages		
Mice, A549, Hela and MDA‐NB‐231 cells	GM‐CSF, LPS, IFNγ, M‐CSF, IL‐4, butylated hydroxyanisole, Apocynin, TEMPO, NAC	FACS, PCR, WB	Lung cancer, breast cancer model	CD11b, CD86, CD163 TNFα, IL‐12, IL‐6, CXCL11, IL‐10, CCL17, CCL18, CCL24, Arginase I, iNOS, F4/8, RELMα, YM‐1, CCL‐2	NOX1 and NOX2 component GTPase RAC1 deletion	Suppressing in M2 macrophages	Late ERK signaling by MAPK pathway	[73]
Nox2^+/y^/Ldlr^–/–^, Nox2^–/y^/Ldlr^–/–^ mice, Bone marrow derived macrophages cells	M‐CSF, (IFN)‐γ	PCR, IHC, ELISA	Abdominal aortic aneurysm	IL‐1β, MOMA‐2, G46, CD68, TNFα, TNF‐β,bFGF, Col 1a1, Fn1, Col4a1, Mmp2, Mmp12, Mmp14, TIMP‐1‐3,iNOS,Arginase‐1,IFN‐γR1‐2,Casp1, Nlrp1a, Nlrp1b, Nlrp3, Hprt 1	NOX2 deletion (gp91^phox^)	Towards M1 macrophages	NF‐κB/AK/STAT1 signaling	[76]
C57Bl6 mice	gp91ds‐tat, scrambled ds‐tat	IHC, IF, WB, FCM	Spinal cord injury	Iba1, CD86, CD206, CD86, iNOS, CD11b	NOX2 inhibition	Towards M2 macrophages	—	[77]
C57Bl6 mice, BV2 microglial cell line	LPS, gp91ds‐tat peptide, scrambled ds‐tat, IL‐4	IHC, ICC, WB	Spinal cord injury	CD206, CD32, CD86, iNOS, Arginase	NOX2 increased in acute condition	Towards M1 macrophages	—	[78]
					NOX4 increased in chronic condition	Towards M1 macrophages		
					NOX2 inhibition	Towards M2 macrophages		
WT and NOX2^−/−^ mice	gp91ds‐tat	PCR, WB, FCM, IHC and IF	Traumatic brain injury	CD11b, TNFα, IL‐1β, IL‐12b, NOS2, IL‐6, Ym1, Arg1, SOCS3, Fizz1, IL‐1RN, IL‐4Rα, TGFβ, CD45, F4/80, gp91^phox^, clic1, CD68, P2Y12, CD16/32, Iba‐1	NOX2 inhibition/deletion	Towards the M2‐like macrophages	IL‐4Rα signaling	[79]
WT, NOX4 knockout (N NOX4^em1cyagen^) and Caco‐2 cells	PMA, LPS, IFN‐γ, Set	IHC, IF, FCM, ELISA	Inflammatory blow disease	CD11c, ZO‐1, Occludin, p‐P65, IL‐1β, TNF‐α, and IL‐6	NOX4 deletion	Suppressed M1 macrophage	NF‐κB signaling	[80]
Human embryonic kidney 293T cells, Hepatoma ML‐1_4a_ cells, Bone marrow‐derived macrophages	Pam3CSK4	ELISA, WB, FCM	Hepatocellular carcinoma	NF‐κB, p65, Arg‐1 and LC3‐I/II, 1.CD204, CD206, CD163, IL‐10, SOCS3, gp91^phox^, TLR2, p62	NOX2 presence	Towards M2 macrophage	TLR2 signaling	[81]
wild‐type (WT) or NOX4^−/−^ mice, PLC/PRF/5 cells, Hep3B cells, THP‐1 cells, bone marrow‐derived macrophages	PMA	PCR, WB, FCM, IHC	Hepatocellular carcinoma	CTGF, Acta2, Ki67, Afp, CD68, Adgre1, MRC1, IL‐6, TNF‐α, IL1B, CD11b, F40/80, CD86, CD206, and Col1A1	NOX4 deletion	Towards M2 macrophage	—	[82]
THP‐1 cell line, RAW264.7 cell line and 8‐week‐old ApoE^−/−^ mice	LPS, N‐acetylcysteine	WB, PCR, IF, ChIP	Atherosclerosis	IL1‐β, IL‐6, CD80, CD86, TNF‐α, H3K27me2, H3K27me3, KDM6A	NOX2 enhanced at transcription level	Towards M1 polarization	KDM6A	[83]
WT and NOX2 KO mice	GM‐CSF, PMA, LPS, Ionomycin	PCR, ELISA, FCM	Japanese encephalitis	CD4, CD80, CD45, CD86, CD44, CD62L, CD11b, INF‐γ, TLR2, CD154, CD25, Ly‐6C, IL‐12, iNOS, TNF‐α, CD206, CXCR3, CD44, CD153, CD25, CD69, CD62L, CCL2, CCL5, and CXCL2	NOX2 deletion	Suppression in M1 macrophages	—	[84]

Helfinger and co‐authors demonstrated that low NOX4 expression reduced the polarization of the M2 macrophages [M(IL4+IL13)] and shifted it towards the M1[M(LPS + IFNγ)], under the influence of NFκB. However, the deletion of NOX4 led to an increase in the expression of NOX2 cells and the concomitant rise in levels of ROS like O_2_^●−^ which was found to encourage the polarization towards M1 macrophages. On the other hand, the deficiency of the NOX2, significantly attenuated the M1 macrophage polarization, without any impact on the M2 macrophages [75]. Han et al. [80] work further explored the mechanism of the NOX4‐mediated macrophages regulation in the inflammatory bowel disease (IBD). Their research findings indicate that the NOX4 deficiency considerably lowered the expression of proinflammatory marker and augmented the expression of Zonula occludens‐1 (ZO‐1), a tight junction protein, and Occludin, a transmembrane protein. Correspondingly, NOX1/4 inhibitor (Setanaxib) treated intestinal macrophages markedly reduced the ROS production and suppressed the M1 macrophages polarization validating the NOX‐mediated regulation [80]. Kigawa et al. [76] investigated the effects of NOX2 deficiency in the abdominal aortic aneurysms (AAA) model. Deletion of NOX2 mitigated ROS production, biased towards the M1 macrophage polarization. Hyperactivation of the M1 macrophages upregulated the expression of IL‐1β and matrix metalloproteinase‐9 (MMP‐9) [76].

Khayrullina et al. [77] demonstrated the M1/M2 dynamics under the influence of NOX2 inhibition in spinal cord injury conditions. Their study concluded that NOX2 inhibitions altered the M1/M2 polarization, shifting towards the M2 macrophages and diminishing M1 macrophage polarization [77]. Bermudez et al. [78] study focused on the role of NOX2 and NOX4 in microglia and their role in macrophage polarization after the spinal cord injury (SCI). The study highlighted that the expression of the NOX isoforms varied over different periods depending on the degree of damage (acute, sub‐acute, and chronic). NOX2 expression increased in acute conditions resulting in ROS production driving the polarization to the M1 macrophages. NOX4 expression was found to be elevated in chronic conditions, encouraging towards the M1 macrophages. M2 macrophage polarization was identified during the acute phase, their expression significantly decreased as the NOX2 expression increased. Similarly, the inhibitions of the NOX2 function shift the polarization towards M2 macrophages. In conclusion, modulation of macrophage polarization promotes cellular homeostasis and enhances the recovery from SCI [78].

Shiau et al. [81] showed that NOX2‐dependent ROS are crucial for hepatocellular carcinoma (HCC)‐induced autophagy and required for the M2 macrophage polarization. This finding contradicts the studies that suggest NOX2 inhibition prevents M2 polarization. This discrepancy can be explained by context‐dependent NOX2 activity and differential signaling pathways. Shiau et al. [81], the research highlighted the pathway via high‐mobility group box 1 (HMGB1), through toll‐like receptor (TLR2)/NOX2 axis and subsequent ROS generation, autophagy, and M2 polarization. Zhao et al. [83] focused on the impact of ROS impact on the epigenetic regulation in macrophage polarization. In this study, the inhibition of ROS synthesis by the N‐acetylcysteine elevated the lysine (K)‐specific demethylase 6A (KDM6A) and promoted the expression of NOX2 at the transcriptional level, and M1 macrophage polarization was favored. When ROS levels were decreased, epigenetic changes occurred, particularly the demethylation of H3K27me3 at the NOX2 promoter, which boosted NOX2 expression and the generation of inflammatory cytokines (IL‐1β, IL‐6, and TNF‐α), which are hallmarks of M1 macrophages. Therefore, it is concluded that the reduction in the ROS increased the NOX2 transcription, enhancing the ROS synthesis, and creating a feedback loop that amplified the pro‐inflammation macrophage condition [83]. Kim et al. [82] investigated the effects of NOX‐4‐mediated ROS effects on the HCC in the setting of liver fibrosis. In this study, the effect of deletion of NOX4 was studied under in vitro and in vivo conditions. NOX4 deficiency caused a notable rise in M2 macrophage polarization, suppressed liver fibrosis, and concurrently indicated a positive contribution towards the advancement of HCC [82].

Choi et al. [84] observed that the NOX2 knockout mice upregulated the macrophage differentiation in the M1 macrophages in response to the viral invasion and hindered the viral replication. This study underscores the pivot role of macrophage phenotype adaptation in controlling viral infection and inflammation [84].

The correlation between ROS and their level of influence on macrophage polarization is crucial. Some studies investigated the effects of NOX1, NOX2, and NOX4 on the polarization from M0 to M1/M2macrophages. Regulation of macrophage polarization is controlled by the up‐regulation and down‐regulation of NOX2 and NOX4, influenced by the ROS. However, the Rac1 and specific subunits of NOX1 and NOX2 are essential for the regulation of macrophage polarization. Yet other detailed studies must be designed to understand how NOX complexes/their specific subunits are involved in macrophage polarization are responsible under different experimental settings. Exploring the functional aspects of NOX subunits under inflammatory conditions could be a promising tool for preventing the onset and progression of disease‐associated inflammation. Note, understanding the molecular mechanism of this macrophage polarization could be a potential therapeutic tool for the treatment of cancer, inflammatory disease, and many other disorders.

## 3. Reactive Oxygen Species (ROS) and Its Relevance

### 3.1. ROS and Their Key Functions

ROS are distinct molecules with oxidizing properties affecting the intracellular redox environment [87, 88]. They can cause oxidative alteration of cellular molecules through the oxidation‐reduction process [89]. ROS are known to have a diverse role in the cellular compartment by maintaining homeostasis and distorting cells that undergo any malfunction [90]. In addition to their crucial role in the growth, activation, and differentiation of B cells, ROS contributes to development, stem cell differentiation, and management of their self‐renewal capacity [91, 92]. ROS are known to serve the cell compartment in various ways such as cell signaling, transcription factor, epigenetic control, circadian rhythm, wound healing, and proteostasis [93]. The alteration of gene expression is also governed by various ROS, such as the nitrogen radical in the nervous and vascular system [94]. In some cases, such as conditions of ischemia and hypoxia, ROS‐mediated signaling helps in mandatory acclimatization for survival. However, increased ROS production results in cell damage by activating the humoral and cell‐mediated response [95]. ROS‐mediated cell damage in association with the immune system contributes to the clearance of pathogens. Production of ROS beyond a certain limit remarkably declines the antioxidant system, consequently detrimental effects on cellular biomolecules, including genetic material, linking ROS to the onset of numerous diseases [96, 97]. To mitigate these effects, it is crucial to understand the pathways involved in their activation and identify the key regulators that control their function.

### 3.2. The Pathway Involved in the ROS Formation

The signal from extrinsic and intrinsic sources is responsible for the production of ROS in the biological system [89]. Extrinsic sources of ROS include analgesic drugs, pollutants, xenobiotics, radiation, and heavy metal ions [98]. On the contrary, the intrinsic sources are the membrane‐bound organelles, such as mitochondria, peroxisomes, the endoplasmic reticulum, and the Golgi complex [99]. Some key enzymes, such as NOX, xanthine oxidase (XO), cycloxygenases (COX), lipoxygenases (LOX), MPO, and nitric oxide synthases (NOS), are actively involved in the ROS production [100, 101]. Mitochondria are a major source of cellular ROS in many tissues; however, in specialized cells such as neutrophils, NOX2 is the predominant ROS‐producing system upon activation [102]. Precisely, about 0.2%–2% of O_2_ are reduced to O_2_^●−^, thus forming H_2_O_2_ and hydroxyl radical (HO^●^) [102, 103]. The mitochondrial electron transport complexes I and III are essential components in the generation of O_2_^●−^ and H_2_O_2_ [104]. The proportion of mitochondria for ROS generation is regulated by the reduction potential of the nicotinamide adenine dinucleotide (NAD) and ubiquinone pool [105, 106]. In the endoplasmic reticulum, the protein disulfide isomerase (PDI) enzyme plays an essential role in protein folding by facilitating the formation and rearrangement of disulfide bonds [107]. PDI enzymes are responsible for the generation of ROS species by transferring electrons from protein molecules to O_2_ [108]. The peroxisome is a single membrane‐bound organelle, a site for several metabolic pathways such as hexose monophosphate shunt, oxidation of fatty acids, amino acid β‐oxidation, breakdown of amino acids, etc. [109]. Acyl CoA oxidase, diamine oxidase, and polyamine oxidase enzymes are also known to be involved in these metabolic pathways that produce H_2_O_2_. Other enzymes such as NOS, D‐aspartate oxidase, and pristanoyl‐CoA oxidase also contribute to the production of different ROS and RNS [110, 111]. Although there are numerous organelles and enzymes, mitochondria and NOX are the main source of inducing ROS [112].

### 3.3. Types of ROS and NOXs in Cells

NOX is a multi–subunit complex expressed in different regions of the cell, solely devoted to ROS production [113]. NOX1 expression has been predominantly detected in colon epithelium, while low expressions are observed in endothelial cells, uterus, placenta, prostate, and brain cells (neurons, astrocytes, and microglia) [114, 115]. The NOX1 activation subunits contain NOXO1, NOXA1 (homologs of p47^phox^ and p67^phox^), and Rac1. The O_2_^●−^ is generated primarily by NOX1, while H_2_O_2_ is considered the key signaling molecule in NOX‐1 mediated signal transduction [115, 116]. The O_2_^●−^ is essential for microbial destruction in neutrophil phagosomes, mainly because of its interaction with MPO. When neutrophils are activated, NOX2 produces O_2_^●−^, which quickly transforms into H_2_O_2_. The production of HOCl, a strong antibacterial oxidant that efficiently eliminates microorganisms, is then catalyzed by MPO using H_2_O_2_. Furthermore, MPO can directly combine with O_2_^●−^ to create oxymyeloperoxidase, which increases the catalytic activity of the latter. This process affects oxidative stress and inflammatory reactions by adjusting the ratio of chlorination to peroxidation [11]. NOX1 isoforms have been found to have a 60% sequence identity with the NOX2 enzyme [57]. NOX2 is also referred to as phagocytic NOX due to its expression primarily in phagocytes, the components of the immune system [117]. For microbial elimination, NOX2 generates O_2_^●−^, which is converted to H_2_O_2,_ which eventually gives rise to HO^●^, when it interacts with the metal ions. H_2_O_2_ interacts with chloride ions and produces HOCl in the presence of the MPO enzyme [118, 119]. Additionally, the O_2_^●−^ generated by NOX2 interacts with nitric oxide (NO) and thus generates peroxynitrite [115].

NOX3 expressions have been reported at the transcription level and are found in the neonatal kidney, head, lung, and spleen [120, 121]. However, NOX3 is expressed mainly in the inner ear and regulates the auditory function by producing O_2_^●−^ [120]. In comparison, NOX4 is a distinctive isoform among the NOX family, expressed mainly in the kidney and blood capillaries [122]. Furthermore, NOX4 is present in the brain (neurons), liver (hepatocytes), keratinocytes, and osteoclast cells. Like other NOX isoforms, NOX4 produces O_2_^●−^ and H_2_O_2_, while their ROS production is sensitively distinct due to the extended E‐loop [123]. NOX5 is the last isoform in the NOX family [57] and is abundantly detected in the spleen, lymphatics, and testes, while its traces are also found in spermatocytes, oligodendrocytes, and cancer cells [124, 125]. In human cells, NOX5 is present in five different types of splice variants, such as Nox5‐α, β, δ, γ, and Nox5‐Short [126].

DUOX1 and DUOX2 are known as thyroid oxidases involved in the production of H_2_O_2_ [127]. They are expressed primarily in the thyroid and respiratory airways and are involved mainly in the biosynthesis of thyroid hormones and contribute to the innate immune‐mediated defense mechanism [128]. De Deken et al. [129] discussed the role of DUOX in immunity, signaling cascades, and the healing process and described the impact of its dysregulation. ROS generated by DUOX are involved in killing invading bacteria and in chemoattraction. DUOX1’s contribution to wound healing via EGFR‐mediated signaling has been validated in airway epithelial cells. The study was conducted in organisms where DUOX‐derived redox signaling is recognized in wound healing, such as leukocyte recruitment. Additionally, H_2_O_2_ production through DUOX creates a positive feedback loop that facilitates receptor signaling activation [129]. Heppner et al. [130] demonstrated that H_2_O_2_ derived from NOXs, DUOX1 and NOX2 modulates redox‐sensitive proteins and affects the EGFR and Src pathways in the airway epithelium. Gu et al. [131] investigated the roles of DUOX1 and DUOX2 in the synthesis of Nicotinic acid adenine dinucleotide phosphate (NAADP). Subsequently, NAADP participates in calcium signaling and T‐cell activation [131]. This points to the fact that NOX isoforms expressed in various locations in the human system generate ROS to mediate cellular functions essential for maintaining whole‐body homeostasis.

Other reactive species including hypohalous acids (HOCl and HOBr), peroxynitrite (ONOO^−^), and chloramines have important roles in NOX enzyme activation, mostly through their impacts on oxidative stress modulation and redox signaling. O_2_^●−^ and NO react to form peroxynitrite (ONOO^−^), which can oxidize proteins and lipids and activate the NOX enzyme. It alters the signaling channels that control the function of immune cells, hence intensifying inflammatory reactions. MPO in neutrophils produces hypohalous acids, which are oxidants that aid in the killing of pathogens and can activate NOX enzymes by changing the cellular redox balance. Particularly, HOCl has been demonstrated to activate NOX2, which encourages the formation of ROS in immune cells. Chloramines are formed when hypohalous acids combine with amines. By influencing protein thiol oxidation, chloramines can modify NOX activity, resulting in increased production of ROS and immune cell activation. Together, these oxidants affect NOX enzyme function, which supports inflammation, oxidative stress management, and immunological defense [132, 133].

### 3.4. Macrophage Regulation by NOX Generated ROS

**Figure 4:**
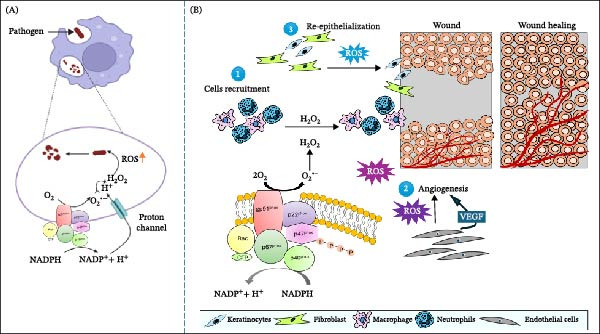
Schematic illustration of the role of ROS in pathogen elimination (left panel A) and wound healing (right panel B). (A) Following pathogen infection, NOX becomes activated and produces ROS (O_2_^●−^ and H_2_O_2_). Elevated ROS are involved in the destruction of pathogens either by inducing oxidative stress or triggering signaling mechanisms to recruit other inflammatory cells that mediate pathogen elimination at the site of infection. (B) NOX‐induced ROS promote wound healing by promoting fibrin deposition (hemostasis), inflammation (involving activation of inflammatory cells), cell proliferation (involving proliferation, migration, and differentiation of fibroblasts and keratinocytes, as well as angiogenesis), and tissue remodeling (through collagen synthesis, cross linking, and strengthening).

Monocytes and macrophages are the main components of the immune system, which is crucial for protection against pathogens [134] (Figure 4A). Macrophages regulate tissue regeneration, initial development, wound healing, and maintaining cellular homeostasis [135]. If there are discrepancies in their function, then it is likely to cause respiratory, hepatic, intestinal, cardiovascular, and neurological diseases [136]. Macrophage functions are mainly governed by ROS primarily generated by the cytosolic NOX and electron transport chain in mitochondria [137, 138]. Furthermore, other enzymes such as XO and MPO contribute to ROS production in macrophage cells [119, 138]. The NOX family releases the O_2_^●−^, which is subsequently converted into H_2_O_2_. Mitochondrial components like the electron transport chain, monoamine oxidase, and cytochrome c activity participate in the formation of ROS [137].

ROS are involved in various cellular functions, including macrophage polarization. ROS, such as O_2_^●−^ and H_2_O_2,_ play a crucial role in macrophage polarization. Dohi et al. [139] studied the impact of NOX‐generated ROS (mainly O_2_^●−^) on the mice model of TBI. TBI led to increased expression of NOX2 subunit, gp91^phox^, thereby elevating levels of ROS, which in turn promote excess production of pro‐inflammatory cytokines, indicating the M1 macrophage polarization. Furthermore, O_2_^●−^ induces the generation of other ROS, resulting in intensifying oxidative stress under TBI conditions [139]. Ming et al. [140] study reported an increase in the production of O_2_^●−^ and H_2_O_2_ in mitochondria and upregulation of the proinflammatory macrophage response. He et al. [141] study demonstrated that the Cu‐SOD and Zn‐SOD mediated H_2_O_2_ modulated the macrophages’ polarization towards the M2 phenotype. Zhang et al. [73] study reported that during monocytes to macrophages differentiation, O_2_^●−^ is produced, and initiates the biphasic ERK signaling. ROS abolition interrupted M2 macrophage differentiation without affecting M1 macrophage polarization. Intriguingly, consistent administration of ROS inhibitors prevented the macrophage’s polarization into the TAMs and suppressed the tumor progression [73]. The work of Griess et al. [142] highlighted the key importance of the ROS and their effects on M1–M2 polarization. In comparison to M1, M2 macrophages have a lower level of ROS and therefore lower level of extracellular H_2_O_2_ production. M2 macrophages, however, required ROS for polarization evident from the fact that the addition of exogenous H_2_O_2_ increased the M2 marker, suggesting its role in the favoring the M2 macrophage polarization [142]. Chen et al. [143] investigated the effects of Oligo‐Fucoidan, a free radical scavenger, on macrophage polarization. Scavenging of ROS such as H_2_O_2_, favored M1 macrophage polarization and repolarized M2 macrophages to the M1 macrophages [143] (Table 3). A recent study by Jin et al. [144] found that the inhibition of NOX4 function by the treatment with apocynin suppressed the H_2_O_2_ production and upregulated the expression of M2 macrophage markers. Suppression of the NOX4‐mediated ROS has been shown to promote the macrophages’ polarization from M1 to M2 [144]. These studies demonstrated the effects of ROS in shifting macrophage phenotypes, and their responses are critical in disease development. Understanding the relationship between ROS production and the shifting of the M1–M2 phenotypes has become an emerging topic of interest. This research could lead to the development of new therapeutic molecules to modulate ROS levels.

**Table 3:** Role of different ROS on proinflammatory and anti‐inflammatory state of macrophages.

Cell line/model organism	Molecules	ROS	ROS source	Studied marker/protein	Response/signaling	Macrophages	Refs.
BV‐2 microglial cell line and mice	IFNγ, IL‐4, IL‐10	O_2_^•−^ ONOO‐	NOX	TNFa, gp91^phox^, iNOS	—	M1 Macrophages	[139]
Bone marrow derived macrophages from Arg‐II^−/−^ mice	Induced by Arg‐II	O_2_^•−^ H_2_O_2_	Mitochondria	TNFα, IL6, and MCP1	Proinflammatory	M1 macrophages	[140]
Alveolar macrophages	Cu, Zn‐SOD	H_2_O_2_	Mitochondria	Ym1 and FIZZ1	regulation of STAT6	M2 macrophages	[141]
	Cu, Zn‐SOD + PEG‐CAT asbestos	H_2_O_2_	Mitochondria	TNF‐α and iNOS		M1 macrophages	
Human primary monocytes cells and bone marrow derived macrophages from mice	Induced by M‐CSF or GM‐CSF and inhibited by butylated hydroxyanisole (BHA)	O_2_^•−^	NOX	CD163, IL‐10, CCL17, CCL18, CCL24	MAPKs, ERK, JNK and p38	Block M2 macrophages	[73]
Peripheral blood mononuclear cells	Induced by GM‐CSF and M‐CSF	H_2_O_2_	NOX	CD163, IL‐10 and CD206	IL‐4 signaling, which activates Stat3	M2 macrophages	[142]
	Induced by GM‐CSF and M‐CSF	H_2_O_2_	NOX	TNFα, IL12b		M1 macrophages	
THP‐1 monocytes	Oligo‐Fucoidan	O_2_^•−^	Mitochondria	—	—	M1 macrophages	[143]
BPH‐1 cells	Apocynin	H_2_O_2_	NOX4	Upregulate MRC1 and IL10	AR/TGF‐β pathways	M2 macrophages	[144]

In the phagosomes, NOX cytosolic subunits produce ROS, which creates a powerful ROS environment for the destruction of microbes [145]. In vitro studies revealed that deleting NOX2 in the macrophages derived from the peritoneal and bone marrow directly hampered ROS production and affected bacterial elimination [146, 147]. In vivo studies demonstrated that mice deficit in NOX1 and NOX2 showed increased parasite load (*Toxoplasma gondii*) [148]. Intriguingly, some studies reported that parasite activity can suppress NOX2 function in the phagosome, ultimately influencing ROS production [149].

NOX enzymes are key players in wound healing, and ROS generated by NOX are also known to contribute to wound healing [150]. Although ROS derived from NOX are key regulators of various cellular processes such as differentiation, proliferation, apoptosis, migration, and contraction, NOX‐derived ROS are especially involved in wound contraction and in the pathophysiology of chronic wounds, such as diabetic ulcers. Since oxygen supply and ATP production are essential for wound healing, NOX activity contributes significantly to these processes. Individuals with chronic granulomatous disease (CGD), a primary immunodeficiency disease, have been reported to be susceptible to infection and have poor wound healing due to NOX impairment [151, 152]. Furthermore, active NOX triggers H_2_O_2_ production, which initiates NF‐κB signaling cascades and increases pro‐inflammatory control [153]. NOX1, which is responsible for the angiogenic properties, upregulated the VEGF receptors [154]. A study demonstrated that the elimination of NOX4 mice reduced HIF1α expression and prevented the repair of cutaneous wounds [155]. Wound re‐epithelialization is also mediated by NOX4‐mediated ROS [156, 157] (Figure 4B).

## 4. Biomolecules Oxidation/Protein Modification by Generated ROS

Biomolecules such as carbohydrates, proteins, lipids, and nucleic acids constitute the basic structural and functional components of all living organisms [158]. Several extrinsic and intrinsic factors cause the generation of a wide variety of ROS (primarily O_2_^●−^, H_2_O_2_, and HO^●^), which in turn cause oxidative modification of different biomolecules [159, 160]. For instance, lipid peroxidation occurs when ROS attack the membrane lipids, particularly polyunsaturated fatty acids. This results in the breakage of carbon–carbon double bonds and the formation of lipid radicals, which eventually promote oxidative stress and cell destruction [161–163]. Indeed, H_2_O_2_ is essential for many biological processes including cell proliferation and differentiation, either directly through the oxidation of the target protein or indirectly through peroxiredoxins. Lipid peroxidation generates chemically reactive aldehydes such as 4‐hydroxynonenal, propenal, malondialdehyde, and heptaldehyde [160, 164].

Protein carbonyls constitute the most predominant oxidative products of proteins due to the oxidation of the amino acid molecules [165]. Moreover, protein carbonyl could also be generated by protein glycosylation as well as the reactions of aldehydes with protein nucleophilic sites [166]. Additionally, intracellular protein modifications occur through indirect methods by the oxidation of lipids or carbohydrates. The key alteration reaction involved in Michael’s addition involves binding of α, β‐unsaturated carbonyl molecules from lipid peroxidation, such as 4‐hydroxy‐nonenal, to amino acid residues, specifically lysine, cysteine, or histidine residues. Other amino acid residues, such as lysine and arginine, interact with glyoxal and malondialdehyde [167–169]. On the other hand, protein carbonylation, where the side chain of non‐aromatic amino acids such as lysine, proline, and arginine is involved in protein crosslinking for managing the structure of the cell [170, 171].

Nucleic acids such as DNA and RNA are also highly prone to oxidative modification by ROS, forming about 30 different types of nucleobases [172]. Oxidative modification of nucleic acids leads to aberration of the purine/pyrimidine nucleosides, inter‐/intra‐strand cross‐linking, and formation of protein and DNA adducts [173]. Nucleic acid oxidative modification is measured by the 8‐oxoGua and 8‐oxodG species [174]. ROS‐induced oxidation leads to the accumulation of DNA adducts, known as secondary DNA products, increasing the risk of genetic destruction and alteration [175]. Understanding biomolecular oxidation is crucial because many diseases are characterized by an enhanced oxidative stress burden. Oxidized proteins are being increasingly used as biomarkers to diagnose, monitor, and predict the severity of certain disease conditions [176]. By identifying and quantifying specific oxidized proteins, researchers and clinicians can gain insights into the underlying mechanisms of the disease, enabling early intervention and the development of targeted therapies.

## 5. Involvement of NOX in Human Diseases

### 5.1. Cardiovascular Diseases

**Figure 5:**
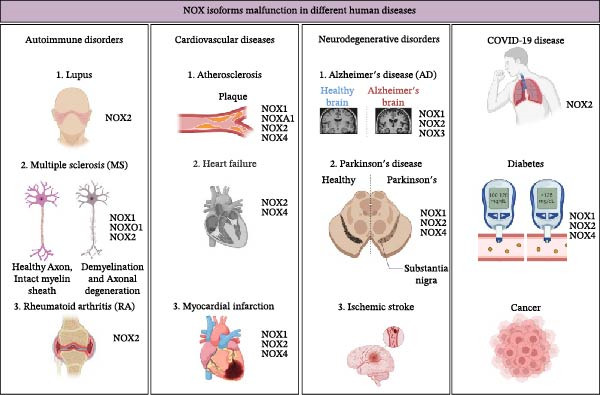
NOX expression in various disease conditions including cardiovascular diseases (e.g., atherosclerosis, myocardial function), neurological diseases (e.g., Alzheimer’s disease [AD], Parkinson’s disease [PD], and Ischemic stroke), autoimmune disorders (e.g., rheumatoid arthritis [RA] and multiple sclerosis [MS]), and diabetes, cancer, and COVID‐19.

In the cardiovascular system, ROS‐mediated signaling helps perpetuate cellular function and their dysfunctions persuade and advance the oxidative stress. In this circumstance, NOX is considered a major contributor to ROS generation [177]. Furthermore, distinct NOX isoforms are associated with the progression of numerous cardiovascular diseases, as described in later sections [178, 179] (Figure 5).

#### 5.1.1. Atherosclerosis

In cardiovascular tissues, predominantly expressed NOX isoforms such as NOX1, NOX2, and NOX4 maintain cellular redox homeostasis [180]. However, the aberrant function of NOX directs the cardiovascular environment toward oxidative stress and triggers atherosclerosis by altering endothelial function, pulmonary hypertension, vascular inflammation, and remodeling [178, 181]. The expression of NOX1 or its subunit NOX activator 1 (NOXA1) and NOX2 was upregulated in atherosclerotic lesions [182]. Additionally, the NOX1/ApoE double‐knockout mice had considerably fewer atherosclerotic lesions in comparison to single‐knockout mice (ApoE^−/−^) [183]. In contrast, NOX4 transcription levels were reduced in mouse plaque, and NOX4 deletion reduced plaque formation. Researchers have also investigated the pro‐atherogenic effects of NOX5; NOX5 did not have a positive effect on the promotion of atherosclerosis but increased the development of aortic aneurism in mice [184, 185]. Interestingly, NOX5 deletion was shown to aggravate atherosclerotic plaques in rabbits [186]. Conclusively, NOX1 and NOX2 have deleterious effects on atherosclerosis, NOX4 had a favorable impact, and the effects of NOX5 are still unclear.

#### 5.1.2. Myocardial Infarction (MI)

ROS inhibit fibroblast proliferation, collagen synthesis, and matrix metalloproteinase activation during heart failure. This results in ventricular hypertrophy, fibrosis, and necrosis contributing to the endothelial and myocardial dysfunction [187]. Several studies confirmed that NOX4 expression was elevated in the heart within 14–28 days after the transverse aortic constriction or addition of phenylephrine or angiotensin II [188–191]. Furthermore, infusion of the lower dose of angiotensin II, which has no effect on alteration of blood pressure, demonstrated defense against cardiac hypertrophy by reducing ROS production under NOX2 elimination. In mice, contraction‐induced pressure overload led to the production of O_2_^●−^ via activation of the NOX4, consequently cardiac malfunction, fibrotic scarring, and apoptosis [192]. Cardiac arrhythmia is a medical condition directly related to elevated ROS levels [179]. Research carried out on the porcine model showed that NOX4 expression increased after the MI, which eventually caused the arrhythmia condition. However, reduction in both pressure and volume in the left ventricles lowered the rate of arrhythmia and NOX2 levels [193]. ROS derived from NOX isoforms also contribute significantly not only to MI but also to myocardial ischemic‐reperfusion (I/R) damage [194].

### 5.2. Neurological Disease

#### 5.2.1. Ischemic Stroke

Ischemic stroke is a neurological disorder characterized by blockage in the blood vessels of the brain. This results in low blood flow and thereby low oxygen supply to the brain, resulting in cell death. This condition leads to enhanced penetrability of the blood–brain barrier (BBB) due to its damage, which leads to further brain injury [195]. The members of the NOX family, and the ROS produced by them, are associated with the disruption of the endothelial barrier and exudation of the BBB [196]. Several previous reports have validated the role of NOX isoforms (e.g., NOX1, NOX2, NOX4, and NOX5) on the onset and progression of ischemic stroke (Figure 5). NOX2 has been recognized as a contributor to BBB dysfunction and its targeted knockout prevented BBB leakage in a mouse model of transient middle cerebral artery occlusion (tMCAO). Also, knockout mice showed decreased MMP‐9 activity, and substantially minimized the lesion size [197]. NOX4 is the prevalent isoform in regulating neurological inflammation, and its expression rises following ischemic stroke [198, 199]. NOX4 knockdown prevented BBB leakage, neurological apoptosis, and decreased oxidative stress in the cell [200]. Like NOX4, NOX5 expression increased ROS production, leakage of the BBB, and induced cerebral deficits in elderly mice. Contrarily, NOX1 expression had a less noticeable and no adverse impact on neurological destruction and lesion volume [200–203]. In conclusion, deletion or inhibition of NOX2, NOX4, and NOX5 functions facilitates the healing process following an ischemic stroke, indicating the key role of NOX isoforms in ischemic stroke.

#### 5.2.2. Alzheimer’s Disease (AD)

Accumulating evidence suggest that the expression of NOX isoform is linked to some neurodegenerative diseases such as AD and Parkinson’s disease (PD) [204]. AD is characterized by the loss of memory and cognitive function. NOX activation and subsequent ROS production induce astrocyte proliferation, neuronal disruption, and inflammation in microglia, contributing to the progression of AD. Additionally, deposition of the amyloid‐β emblematized the commencement of AD. Different varieties of amyloid beta engaged in the activation of different NOX isoforms (e.g., NOX1, NOX2, NOX3, NOX4, and NOX5) resulted in neuronal damage and neuroinflammation [204–206]. Among them, NOX 2 and NOX4 isoforms have been reported as the main contributors to the AD pathology. In AD mice models, NOX2 deletion did not induce oxidative stress and showed no evidence of cerebral vascular impairment, even with a specific proportion of amyloid‐β peptide [207]. NOX4‐mediated oxidative stress compromised mitochondrial metabolism by encouraging lipid peroxidation and ferroptosis in astrocytes of AD [208].

In addition to oxidative stress caused by NOX, mitochondrial dysfunction is also responsible for AD pathogenesis. Mitochondria produce a significant amount of ROS; an imbalance in oxidative homeostasis further enhances ROS production, leading to increased lipid peroxidation, oxidation of biomolecules, and neuronal damage. Impaired mitochondrial function in AD also reduces electron transport chain activity and citric acid cycle enzyme activity, resulting in hypometabolism. Additionally, the interaction of β‐amyloid protein with mitochondrial enzymes increases ROS production, leading to synaptic degradation and cognitive decline. Chronic oxidative stress also exacerbates the hyperphosphorylation of Tau proteins [209]. In summary, mitochondrial dysfunction leads to oxidative stress, impairs energy metabolism, and promotes protein aggregation, all of which contribute to AD disease progression.

#### 5.2.3. PD

Mounting evidence indicates that NOX‐derived ROS play a crucial role in the progression of PD, a neurological disorder characterized by increased oxidative stress and progressive neuronal degeneration. PD is primarily characterized by the loss of dopaminergic neurons [210]. Dopaminergic neurons are enriched in dopamine and have high mitochondrial activity. Dopamine is a highly unstable neurotransmitter that can undergo autooxidation, producing O_2_^●−^ and H_2_O_2_, processes that are enhanced in the presence of NOX‐derived O_2_^●−^ [211]. Tu et al. [212] found that oxidation of dopamine and its by‐products activate microglial NOX2, leading to excessive O_2_^●^− formation. This feedback loop generates excessive ROS and contributes to progressive dopaminergic neurodegeneration [212]. Choi et al. [213] suggested that NOX1 plays a critical role in amplifying intracellular oxidative stress involved in the progression of PD via dopamine‐dependent pathways. NOX1‐driven amplification of oxidative stress directly triggers progressive degeneration of dopaminergic neurons through mitochondrial dysfunction [213].

Various NOX isoforms are distributed across different regions of the brain. NOX1, NOX2, and NOX4 are expressed in neurons and microglial cells, while NOX2 is also detected in astrocytes [210]. In microglial cells, NOX‐derived ROS are responsible for killing invading microorganisms during inflammatory conditions [214]. NOX1 is involved in synaptic plasticity and enhances D2 receptor signaling in indirect pathway medium spiny neurons (iMSNs) in the central striatum [215]. However, NOX2 promotes synaptic plasticity by enhancing NMDAR function and is essential for long‐term potentiation (LTP) and long‐term depression (LTD) in the visual cortex [216].

In PD, expression of the NOX1, NOX2, and NOX4 isoforms was found to be increased [217]. Ma et al. [196] reported that the activities of NOX1 and NOX2 are increased in PD, influencing ROS production. Microglial NOX2‐derived ROS play a key role in dopaminergic neurodegeneration, while NOX1 is responsible for oxidative damage in the striatum [196]. Cristóvao et al. [218] demonstrated that increased NOX1 expression enhances α‐synuclein expression and aggregation in dopaminergic neurons in a PD model. Their study concluded that suppression of NOX1 activity in the substantia nigra reduces dopaminergic neuronal loss and α‐synucleinopathy [218]. Keeney et al. [219] investigated the influence of disease condition on the activity of NOX2 in mice models of PD. Only in chronic PD models, they observed a high NOX2 activity in neuronal and microglial cells, particularly in the substantia nigra. Interestingly, acute and sub‐acute PD models showed elevated NOX2 activity only in neuronal cells but not in microglial cells, implying the critical role of neuronal NOX2 in the progression of PD [219]. Recent findings by Pullara et al. [220] showed a direct correlation between NOX2 activity and calcium signaling in the aggravation of PD. Activation of NOX2 and concomitant rise in ROS have been suggested to disrupt the calcium signaling which in turn leads to progression of PD [220]. The NOX4 isoform that is predominantly expressed in the CNS plays a major role in PD pathogenesis. Previous studies have shown the accumulation of NOX4 in the hippocampus of PD. This rise in NOX4 levels has been correlated with upregulation of the MPO enzyme and osteopontin (OPN) in astrocytes of PD [221]. The findings of Lin et al. [222] highlighted the critical role of NOX4 expression in PD. Upregulation of NOX4 levels has been shown to induce apoptosis of dopaminergic neurons, leading to the aggravation of PD. Further, they reported that inhibition of NOX4 led to a reduction in apoptotic cell death of dopaminergic neurons in animal models of PD [65]. Altogether, it can be suggested that the therapeutic targeting of NOX isoforms might be a possible treatment for the amelioration of PD‐associated pathology.

### 5.3. Autoimmune Disorders

NOX‐derived ROS contribute to persistent inflammation, tissue damage, and immune cell dysfunction in different autoimmune diseases such as RA, systemic lupus erythematosus (SLE), and multiple sclerosis (MS) [223, 224]. The abrogation of NOX2 amplifies the positive impact on SLE progression by reducing inflammation and altering T‐cell function [225]. Neutrophil cytosolic factor (NCF‐1) gene encodes NOX2 complex subunits p47phox/Ncf1. The alteration in the NCF‐1 subunit compromises the defensive function of dendritic cell‐derived ROS, resulting in the onset of SLE [226]. The development of NETs during autoimmunity is critical, and NOX‐derived ROS play an important role in this. NET serves as a source of autoantigens, increasing autoantibody formation, and the NET protein is involved in cell damage and SLE progression [227]. RA is characterized by constant irritation in the joints, causing discomfort, swelling, and possible joint damage [228]. However, depending on the genetic makeup, NOX‐generated ROS have been demonstrated to play a protective function in autoimmune diseases. In mice, mutations in the NCF1 gene resulted in the loss of NCF1 and NOX2 activities, as well as an increase in the autoantigen in arthritis [229]. The induction of the NOX2 subunits p22phox and p47 caused the emergence of a microglial lesion in neural cells in MS [230]. Furthermore, MS patient brain samples showed elevated expression of the NOX1 and NOX organizer (NOXO1) in microglial cells [231].

### 5.4. Diabetes

Diabetes is a condition caused by an increase in glucose and associated ROS generation, causing adverse effects on tissues [232]. Research emphasized the significant contribution of various NOX isoforms (NOX1, NOX2, and NOX4) to ROS production and β‐cell malfunction. The generation of ROS by NOX2 causes insulin resistance in endothelial cells, compromising vascular function under conditions of diabetes. In contrast, NOX2 deletion increased vascular function by decreasing ROS concertation. Under hyperglycemic conditions, ROS production by NOX causes the generation of mitochondrial O_2_^●−^, which is accountable for the onset of diabetic nephropathy [233]. Furthermore, NOX1 expression is promoted, while SOD‐1 activity is decreased, facilitating ROS generation and vascular calcification [234]. The Sma and Mad ‐relatedProtein 3 (Smad3) improve acute kidney injury (AKI) susceptibility in diabetic mice through its interaction with p53, resulting in activation of NOX4‐mediated ROS generation. This pathway aggravates oxidative stress, driving kidney damage, and deteriorating AKI outcomes in diabetes [235].

It is well documented that NOX plays a critical role in the dysfunction of pancreatic beta cells. Pancreatic beta cells express several NOX isoforms, including NOX‐1, NOX‐2, NOX‐4, NOXO1, and NOXA1 [236]. In vitro studies showed that stimulation of human islets, mouse islets, or murine beta‐cell lines with a cocktail of pro‐inflammatory cytokines (TNFα, IL‐1β, and IFN‐γ) significantly induced the expression of NOX‐1. Thus, it can be suggested that the possible role of NOX‐1 activity as an important component to beta cell pathogenesis [237]. Genetically engineered mice with co‐associated increased glucose tolerance and resistance to streptozotocin‐induced hyperglycemia showed a reduction in cytokine‐induced NOX‐1 expression [238]. A connection between NOX‐2 activity and beta cell dysfunction brought on by very low‐density lipoprotein [239] or free fatty acid [240] suggests a pathogenic role for NOX‐derived ROS in beta cells [240].

### 5.5. Cancer

Mounting research has highlighted that NOX isoforms participate in tumor formation, cell proliferation, metastasis, cell invasion, and angiogenesis [3]. Any aberrative expression of the NOX family isoforms or their subunits can substantially affect the cancer behavior [241]. ROS‐mediated NOX4 can indeed influence tumor progression by controlling key signaling pathways [3, 242]. Indeed, NOX family members and their subunit’s expression intensify in various types of tumor tissues, linking them as predominant contributing factors in cancer. The expression of the NOX isoforms (e.g., NOX2, NOX4, and DUOX1) was upregulated in the gastric cancer‐affected individual’s tumor cells. The overexpression of NOX1 in the fibroblast cells leads to the formation of new tumors and promotes their growth despite the constraints of ROS (H_2_O_2_), indicating that high levels of ROS are not efficient in the early phases of tumorigenesis [3, 243].

Dysregulation of different isoforms of NOXs has been reported in multiple cancers. Altered expression of NOXs results in the modulation of cell proliferation and survival, angiogenesis, and metabolic adaptation. NOX1 isoform is one of the most implicated isoforms which is activated during gastric cancers, where ROS increase inflammatory and oncogenic signaling. It also enhances VEGF‐driven tumor progression in colon cancer and promotes cell growth, cytoskeletal remodeling, and angiogenesis in K‐Ras‐transformed cells. Additionally, Nox1 promotes the growth of E6/E7‐altered keratinocytes and aids in the angiogenesis of prostate cancer [244]. In contrast, there is little evidence connecting NOX2 and NOX3 to carcinogenesis, and their involvement in cancer are still mostly unknown [244]. NOX4’s function is more complex in cancer. NOX4 exhibits distinct effects in different forms of cancer. By being significantly expressed in non‐small cell lung cancer tissues, increasing the proliferation of gastric cancer cells via the glioma‐associated oncogene homolog 1(GLI1) pathway, and controlling TGF‐β1‐driven metabolic rewiring in glioblastoma cells, NOX4 has been demonstrated to promote tumor growth. On the other hand, NOX4 inhibits the growth of pancreatic cancer by causing cellular senescence mediated by ROS. Furthermore, it has the ability to influence the immune response and the tumor microenvironment [245]. NOX5, a calcium‐activated isoform, increases cell survival and proliferation in esophageal adenocarcinoma [246]. On the other hand, DUOX1 and DUOX2, which are normally involved in epithelial repair and innate defense, become dysregulated in lung cancer, where they affect cell migration, tissue remodeling, and chronic inflammatory signaling [247]. Together, these enzymes alter the redox environment of tumors, allowing cancer cells to make use of ROS as signaling molecules that encourage the development and spread of malignancy.

### 5.6. Viral Infection

During viral infections, NOX‐mediated ROS are essential for immune modulation because they activate NOX2 through the PKC pathway, which produces H_2_O_2_ [248]. This lowers the antibody concentration and hinders the antiviral response, whereas NOX2 deletion raises IgG levels and modifies the Type 1 Helper T cell (Th1) response. NOX2 expression dramatically rose during COVID‐19 and was correlated with the severity of the illness [249, 250]. In COVID‐19 patients, NOX activation raises tissue factor expression, which encourages thrombotic and blood clotting events. When a virus enters endosomes, NOX‐driven ROS generation is triggered, activating pathways that result in serious vascular problems [251]. Previous studies have reported that RNA viruses can activate the NOX enzyme in vascular endothelial cells. This happens through binding of their single‐stranded RNA to TLRs. Activation of NOX2 facilitates the production of H_2_O_2_, modulates TLR7, and suppresses downstream antiviral and humoral responses. However, in experimental systems, suppression of ROS production attenuated influenza A virus virulence [248]. In human nasal epithelial cells, influenza A virus infection promotes ROS generation through DUOX2. Attenuation of DUOX2 exacerbates viral infection, which establishes the role of DUOX2‐derived ROS in suppressing viral infection [252]. Additionally, NOX1 attenuated lung inflammation and oxidative stress in mice during the onset of influenza A virus infection [253]. In some cases, ROS produced by NOX2 weaken the body’s capacity to control Japanese encephalitis virus (JEV) outside the brain by hindering macrophage polarization toward the M1 state. Consequently, this increases viral replication and invasion, worsening disease progression [84]. NOX activity is enhanced during viral infection, which suppresses antioxidant enzyme activity and ultimately increases ROS generation. Increased ROS levels are linked to viral replication and pathogenesis [254].

Elevated ROS levels and NOX2 activation are associated with severe thrombotic events in COVID‐19 patients. An increased risk of thrombotic complications and the severity of the condition are correlated with high levels of soluble NOX2‐derived peptide (sNOX2‐dp) [250]. This suggests that Severe acute respiratory syndrome coronavirus 2 (SARS‐CoV‐2) may promote viral propagation by inhibiting immune defense by activating NOX2 through a mechanism that is dependent on TLR7. Since COVID‐19 patients have higher levels of NOX2 and NOX5 in their vascular endothelium, NOX activation is also associated with cardio‐microvascular dysfunction [255]. According to Paul et al. [256], inflammation linked to COVID‐19 is exacerbated by pulmonary NOX activation. Endothelial NOX2 is triggered by the cytokine storm, increasing ROS levels and worsening the severity of the disease [256].

### 5.7. NOX2 and Its Role in CGD

CGD is a rare genetic disorder of the phagocytic cells of the immune system. It is caused by loss or mutations in the components of the NOX2 NOX complex. The NOX2 isoform plays an important role in the generation of ROS during respiratory burst in phagocytes (monocytes, macrophages, and neutrophils). Defects in the NOX2 complex lead to reduced production of ROS (O_2_^●−^ and H_2_O_2_) and consequently impaired functioning of phagocytes [257]. As a result, individuals with CGD experience severe bacterial and fungal infections due to defective phagocytic cells.

The majority of CGD manifestations are caused by mutations to the gp91phox, p22phox, p47phox, p67phox, or p40phox genes. These genes are linked to the five components of NOX. The NOX2 variation is the only CGD mutation that is X‐linked recessive; the majority are autosomal recessive. CYBB [cytochrome b (−245), beta subunit], which is found on chromosome X, encodes the NOX2 (gp91phox) gene. The CYBB variation is found in about 70% of CGD patients, which explains why CGD is more common in men. 25% of cases are caused by a guanine‐thymine deletion in exon 2 of the p47phox gene, which is encoded by NCF1. The remaining 10% of cases are caused by other mutations [258].

### 5.8. IBD

IBD is a chronic disorder that gives rise to inflammation in the digestive tract. IBD is commonly induced by environmental stressors and facilitates the generation of a dysregulated inflammatory response toward the microbiota in individuals with genetic susceptibility [259]. Hayes et al. [260] demonstrated that dysfunction of NOX1 and DUOX2 triggers very early onset inflammatory disease (VEOIBD) through compromised ROS generation in the intestinal epithelium. Makhezer et al. [261] revealed that NOX1‐mediated ROS are crucial for mucosal immunity and regulate inflammation by controlling the expression of lipocalin‐2 (LCN‐2) in colon epithelial cells. Inflammatory signaling activates NOX1, facilitates ROS production, and leads to the activation of redox‐dependent pathways, resulting in the stimulation of LCN‐2. In contrast, inhibition of NOX1 function hampers LCN‐2 expression and consequently weakens pathogen defense [261]. Dang et al. [262] in their study described the dual function of NOX‐derived ROS, genetic variation of NOX associated with impairment of the ROS generation in the early onset of IBD. On the contrary, overexpression was also found associated with the disease, indicating the dual role of the NOX in the pathogenesis [262]. Dang et al. [262] also described the dual function of NOX‐derived ROS. Genetic variations in NOX associated with impaired ROS generation have been observed in early‐onset IBD [259].

### 5.9. Heparin‐Induced Thrombocytopenia (HIT) and NOX

HIT is a lethal and life‐threatening condition that arises due to complications of heparin administration. The characteristic features of HIT include a reduction in the platelet count and a hypercoagulable state. Research has shown that NETs, produced via NETosis, are key contributors to thrombosis in HIT through activated neutrophils [263]. Notably, NET formation depends on ROS generation, which acts as a critical mediator of NETosis. NOX2 is the key enzyme involved in ROS production that facilitates the process of NETosis. ROS produced by NOX2 are disseminated throughout the thrombus, stimulating both platelets and neutrophils and thereby intensifying the thrombotic condition. However, blocking ROS production in neutrophils impairs NETosis and thrombus formation. Additionally, inhibition of NOX2 by diphenyleneiodonium chloride or GSK2795039 significantly reduces NETosis and thrombosis without affecting thrombocytopenia in animal models of HIT [263]. Collectively, these results highlight that suppression of NOX2 markedly reduces the high‐risk thrombosis associated with the HIT state.

In summary, NOX2 is a key contributor to the prothrombotic process in HIT, linking immune complex activation to neutrophil‐mediated thrombosis, and targeting NOX2 could be a promising strategy for preventing thrombosis in HIT.

### 5.10. NOX and Brain–Spine Injury

SCI and TBI are major CNS injuries caused by external forces and frequently co‐occurring conditions. SCI mainly affects the spinal cord, resulting in motor and sensory loss, while TBI causes brain injury, leading to emotional and physiological difficulties [264]. In CNS injury, ROS derived from NOX are key contributors to inflammatory mediator release after traumatic injury [265]. Schiavone et al. [266] suggest that NOX2‐derived ROS contribute to interneuron loss and disrupt inhibitory circuitry after TBI. Laabei et al. [267] investigated the impact of NOX attenuation on post‐injury outcomes in TBI. NOX2 acts as an initiating signal for NLRP3 inflammasome activation; inhibition of NOX2 modulated downstream inflammasome signaling. As a result, the inhibitory treatment showed mild improvement in neurological function after TBI [267]. Cooney et al. [268] studied the impact of NOX inhibition on post‐injury inflammation after SCI. Inhibition of NOX2 reduced the production of pro‐inflammatory cytokines and the expression of oxidative stress markers in the SCI condition [268]. Khayrullina et al. [269] demonstrated the role of NOX2 and NOX4 in ROS production after injury. Deletion of NOX2 improves functional recovery, neuroprotection, and anti‐inflammatory phenotypes, while a single dose of NOX4 inhibition does not improve long‐term recovery [269].

### 5.11. Treatment Targeting the NOX and ROS

NOX enzymes, known for ROS production, play a crucial role in several cellular processes. However, hyper‐ or excess ROS generation promotes disease, as discussed in the above section. Under these conditions, the need for non‐specific antioxidants shifts toward specific NOX inhibition. Liao et al. [270] demonstrated the effects of Setanaxib, which specifically inhibits NOX1 and NOX4 activity. Targeting NOX1 and NOX4 modulated apoptosis, senescence, and oxidative stress pathways, reducing alterations in retinal structure and function induced by retinal ischemia‐reperfusion [270].

Xiong et al. [271] discussed several inhibitors, including Diphenyliodine (DPI), Apocynin, S17834, NOX2ds‐tat, ML171, NOS31, LMH001, Celastrol, and Ebselen. DPI was the first inhibitor used to inhibit all NOX isoforms, engaging in irreversible binding with flavin adenine dinucleotide (FAD). Apocynin specifically inhibits NOX2 in the presence of MPO. S17834 reduced endothelial NOX activity without affecting O_2_^●−^ production through XO. NOX2ds‐tat was developed specifically to target NOX2 and attenuate NOX2‐mediated ROS production provoked by several stimuli. ML171, a selective NOX1 inhibitor, also has some effects on other NOX isoforms. Celastrol, a bioactive‐derived inhibitor, is effective toward NOX1 and NOX2. Ebselen inhibits NOX2 by suppressing H_2_O_2_ and other hydrogen‐catalytic functions. NOS31 targets the inhibition of NOX1 activity. Recently designed LMH001 interacts with exposed SH3 active sites, suppressing NOX2 initiation [271]. Sylvester et al. [272] briefly outlined clinical trials aimed at attenuating NOX isoforms in various diseases. Setanaxib, a NOX inhibitor, has been evaluated in clinical trials for chronic fibrotic and inflammatory diseases, confirming their efficacy and safety. Additionally, NOX2ds‐tat inhibitors have shown disease‐modulating effects in preclinical models of several conditions, such as diabetic, cardiac, and pulmonary fibrosis [272].

## 6. Conclusion

ROS play crucial roles in cellular physiology and pathological functions. In the cellular system, several ROS are produced within mitochondria as well as by some enzymes, including NOXs. Mounting research evidence shows that ROS generated through the catalytic action of NOXs are critical for cell differentiation, phagocytosis, wound healing, and monocyte‐to‐macrophage transitions. The two vital processes of the immune system such as monocyte differentiation and macrophage polarization are interlinked with alterations in NOXs expression and subsequent change in ROS levels. Several studies demonstrated that the macrophage polarization (M1 and M2) is mainly governed by different NOX isoforms such as NOX1, NOX2, and NOX4 and associated ROS dynamics. Despite its complexity, the immune system maintains the dynamic equilibrium between NOX and ROS. However, any imbalances in ROS levels modulate the M1–M2 polarization, leading to the onset and progression of several diseases. Through understanding of the underlying molecular mechanisms of NOX‐mediated macrophage polarization reveal valuable insights on how to modulate immune dynamism in inflammatory and anti‐inflammatory conditions. Targeting NOX‐derived ROS pathways could offer powerful potential for the treatment of chronic human diseases, including neurological diseases, cancer, diabetes, and several other inflammatory diseases. Additionally, therapeutic targeting the NOX subunits could offer a potential positive outcome on the modulation of altered ROS commonly seen in the progression of disease conditions. Determining the exact levels of ROS still poses a challenge in both in vivo and in vitro experimental settings. Therefore, developing new experimental setup methods could provide an in‐depth analysis of cellular ROS and enhance our knowledge in relation to NOX.

## Funding

This study is supported by Univerzita Palackého v Olomouci, IGA_PrF_2026_022 (Modern trends in research in general and molecular biophysics).

## Conflicts of Interest

The authors declare no conflicts of interest.

## Data Availability

Data sharing is not applicable. The manuscript does not contain any experimental data. Figures 1–5 in the manuscript were created using a licensed version of BioRender.com.
